# Digital Infrared Thermographic Imaging for Limb Salvage in Patients at Risk of Amputation: Prospective Observational Study

**DOI:** 10.2196/69072

**Published:** 2025-07-21

**Authors:** Víctor Manuel Loza-González, Eleazar Samuel Kolosovas-Machuca, Patricia Aurea Cervantes-Báez, José Luis Ramírez-GarcíaLuna, Edgar Guevara, Mario Aurelio Martínez-Jiménez

**Affiliations:** 1 Doctorado Institucional en Ingeniería y Ciencia de Materiales (DICIM-UASLP), Universidad Autónoma de San Luis Potosí, San Luis Potosí, Mexico; 2 Coordinación para la Innovación y Aplicación de la Ciencia y Tecnología, Universidad Autónoma de San Luis Potosí, Sierra Leona No. 550 Col. Lomas 2da. Sección, Planta Baja, San Luis Potosí, 78210, Mexico, 52 4448262300 ext 8433; 3 Burn Care Unit and Wound Care Center, Hospital Central Dr. Ignacio Morones Prieto, San Luis Potosí, Mexico; 4 Division of Experimental Surgery, Faculty of Medicine, McGill University, Montreal, QC, Canada; 5 Faculty of Science, Universidad Autónoma de San Luis Potosí, San Luis Potosí, Mexico

**Keywords:** infrared thermography, amputation, diabetic foot, traumatic injuries, limb salvage

## Abstract

**Background:**

Scores and prediction models such as the mangled extremity severity score (MESS) for trauma and the Wound, Ischemia, and foot Infection (WIfI) classification for diabetic foot ulcers help in the decision-making process for amputation. However, these tools can be subjective as they depend on the experience of the medical staff applying them.

**Objective:**

This study aimed to assess the impact of temperature measurement using infrared thermal imaging in extremity salvage in patients at risk of limb amputation.

**Methods:**

We included 29 patients who sought a second opinion after an amputation recommendation. Infrared thermographic images were acquired to measure the temperature difference (ΔT) between the injured and uninjured limbs. For patients with salvaged limbs, we provided clinical follow-up for up to 12 weeks.

**Results:**

Of the 29 patients enrolled in the study, 27 limbs were salvaged. Thermographic imaging allowed the distinction into two groups: the first group of 18 patients with mean ΔT value of −3.6 °C (SD 1.99 °C) , and the second group of 9 patients with mean ΔT of 3.36 °C (SD 2.71 °C). None of the patients in either group showed progression in ΔT values within the first 5 days; at the twelfth week, ΔT values approached 0 °C at wound closure. Of the two patients who required amputation, one showed an initial ΔT of −4.3 °C, which worsened to −5 °C by the fifth day, and the other patient showed an initial ΔT of −4.5 °C, which worsened to −5.8 °C by the fifth day.

**Conclusions:**

Digital infrared thermography is a tool that may help guide limb salvage in patients with uncertain clinical diagnoses. This imaging modality allows visualization of thermal differences and patterns derived from thermal changes in patients at risk of limb amputation.

## Introduction

Amputations are performed due to both traumatic and nontraumatic causes, including peripheral vascular disease, diabetes, and cancer. Diabetes and peripheral disease are the leading causes of nontraumatic amputations of the lower extremities [1,2]. Along with its high prevalence, the number of patients with diabetes mellitus is expected to increase to 700 million by 2045. It is estimated that 53% of diabetic patients are at risk of developing diabetic foot, with an augmented risk of amputation resulting from neuropathy, peripheral arterial disease, biomechanical deformity, limited articular mobility, and ulceration [1,3-6]. Following major amputation, the patients are at a higher mortality risk than those with some malignant diseases—up to 70% after 5 years—often dying due to cardiovascular disease [1,7]. While many classification systems are available, the WIfI classification was designed to assist with risk stratification of patients with diabetes. Despite its acceptance, its correlation with stage and clinical outcomes remains questionable, as it does not consider hemodynamic measures of limb ischemia [8].

Amputations related to accidents have decreased in frequency, but the decision to perform an amputation or reconstruct a lower extremity is a complex one for surgical teams. This is supported by the mangled extremity severity score (MESS), which evaluates acute limb ischemia, soft tissue damage, and physiological conditions. This score was developed over 32 years ago; however, medical advancements have raised questions about its current accuracy [9,10]. Hand injuries account for 12% of all traumatic injuries and 90% of amputations in the United States, with fingertip injuries being the most common cause. Despite this fact, the evaluation of patients with finger injuries is done on a case-by-case basis, and no formal clinical practice guidelines with treatment strategies are available for surgeons [11-15].

Body temperature has long been used as a natural indicator of diseases; it can be affected by conditions such as peripheral arterial disease and infections [4]. Medical infrared thermography can objectively measure body temperature changes related to the blood by detecting the long-wave infrared radiation emitted from the body. It creates temperature maps with abnormal regions that are detected and analyzed by the software, comparing regions of interest with the unaffected contralateral regions [16]. Based on the principle that any object with a temperature greater than 5 K emits infrared radiation, infrared thermography can detect heat derived from microcirculation flow 1 to 2 mm below the surface of the skin using a dense grid of sensory pixels [17,18]. It is noninvasive, fast, reliable, cost-effective, contactless, avoids exposure to ionizing radiation, and can produce multiple images within short time intervals without the discomfort of the patients [19,20]. The use of infrared thermography over the years has allowed scanning foot temperature in diabetic patients and evaluating the state of an injured limb to help decide if a patient’s limb must be amputated [17]. It has also been used for screening fever, breast cancer, and diagnosis of vascular disorders by observing blood flow patterns, showing that the skin can reflect the status of the cardiovascular system. In cases of compromised small vessels and unaffected large vessels, thermography could be more effective than ultrasound-based techniques [21-23]. Recent advances have enabled thermography tools to be coupled with smartphones, facilitating the extended use of this technology [22].

### Goal of This Study

Based on the thermal changes caused by blood flow and our previous study which predicted the treatment modalities in burn patients using thermal imaging, we aimed to use infrared thermography in patients at risk of amputation [21]. As wounds have heterogeneous thermographic patterns, measurements were taken from the fingers of the affected limbs and compared with the contralateral side, as finger evaluations have been done previously with devices that evaluate tissue oxygenation and limb blood flow [24]. By analyzing thermal changes between healthy and affected extremities, we proposed a linear regression model to help in the decision-making process by physicians treating such patients. The objective of this study was to determine the impact of digital infrared thermographic imaging for limb salvage in patients at risk of limb amputation by detecting temperature differences between the affected and healthy limbs.

## Methods

### Patients

This prospective cohort study included patients referred to the surgery department at Hospital Central “Dr. Ignacio Morones Prieto,” a tertiary-level hospital in San Luis Potosí, Mexico, from another hospital or wound clinic for a second opinion regarding limb salvage between November 2023 to November 2024. Recruitment was performed during routine clinical consultations by the wound care team. Eligible patients were invited to participate, and informed consent was obtained before enrollment. All included patients had received recommendations for amputation, based on the clinical assessment of traumatic limb injuries or diabetic foot ulcers. Inclusion criteria were patients of any sex, older than 18 years, with a MESS of <7 or WIfI classification of ≤4. Exclusion criteria included patients with frank tissue nonviability, hemodynamic instability, severe wound infection, sepsis, or septic shock. Demographic variables such as the patients’ gender and age were registered and information about the etiology of the lesion, patient comorbidities, anatomical site of the wound, and wound area were recorded.

### Infrared Thermography

Infrared thermography scanning was performed using Flir T600 infrared camera (97070, FLIR System) in a room with a controlled temperature of 22 °C, atmosphere humidity of 40%, and under controlled light conditions. Scanning was performed from a distance of 0.5m at the patient’s bedside. Before each scan, the dressings were removed, and after cleaning, the wounds were dried with sterile gauze. The affected extremities were left uncovered for 20 minutes before scanning The camera was powered on 10 minutes prior to image acquisition to allow for sensor stabilization. Thermographic images were taken following the protocol as proposed in the thermographic imaging in sports and exercise medicine (TISEM) checklist, with skin emissivity set at 0.98. A single expert in thermographic imaging interpreted the images using the FLIR Tools software (version 5.13.18031.2002; Teledyne FLIR) [17,25].

Scanning was conducted on the day 1 of medical evaluation, day 5 after hospital admission, and at the 12 weeks in outpatient follow-up, after complete wound healing. The regions of interest included toes or fingers of both the affected and healthy limbs. The difference in average temperatures (ΔT) of the compared areas between the affected limb and the contralateral healthy limb was calculated and used for the statistical analysis.

### Statistical Analysis

Demographic data are expressed as the mean (SD) or proportions, as appropriate. Statistical analysis was performed using the R software (version 4.1.2; R Foundation for Statistical Computing, including the *R Commander* package (version 2.9‐5). The Shapiro-Wilk test was used to verify the normality of the data distribution, and the nonparametric Friedman test to assess differences across the time points within each group. When the Friedman test indicated significant results, we conducted post-hoc multiple comparisons using the Bonferroni correction to identify specific pairs of time points with statistically significant differences. Linear regression was used to compare the obtained ΔT and identify confounding factors such as sex, age, wound area, evolution time, day of wound closure, diagnosis, comorbidities, and clinical outcome. Wound healing in both groups was evaluated with Kaplan-Meier survival analysis. A *P* value <.05 was considered statistically significant.

### Ethical Considerations

The study was conducted according to the guidelines of the Declaration of Helsinki and was approved by the Research Ethics Committee of the Hospital Central “Dr. Ignacio Morones Prieto” with Cofepris 17 CI 24 028 093 (protocol code 08‐23) at the Department of Surgery and Wound Care. Informed consent was obtained from all subjects involved in the study. The consent form included use of clinical and thermographic imaging data. All data were anonymized prior to analysis no personal identifying information was included in the study database or in any publication materials. Participants did not receive financial or material compensation for their participation in the study. No identifying images of individual participants were included in the manuscript or supplementary material. All thermographic and clinical images were captured in a way that preserved patient anonymity.

## Results

### Demographic findings

**Figure 1.**
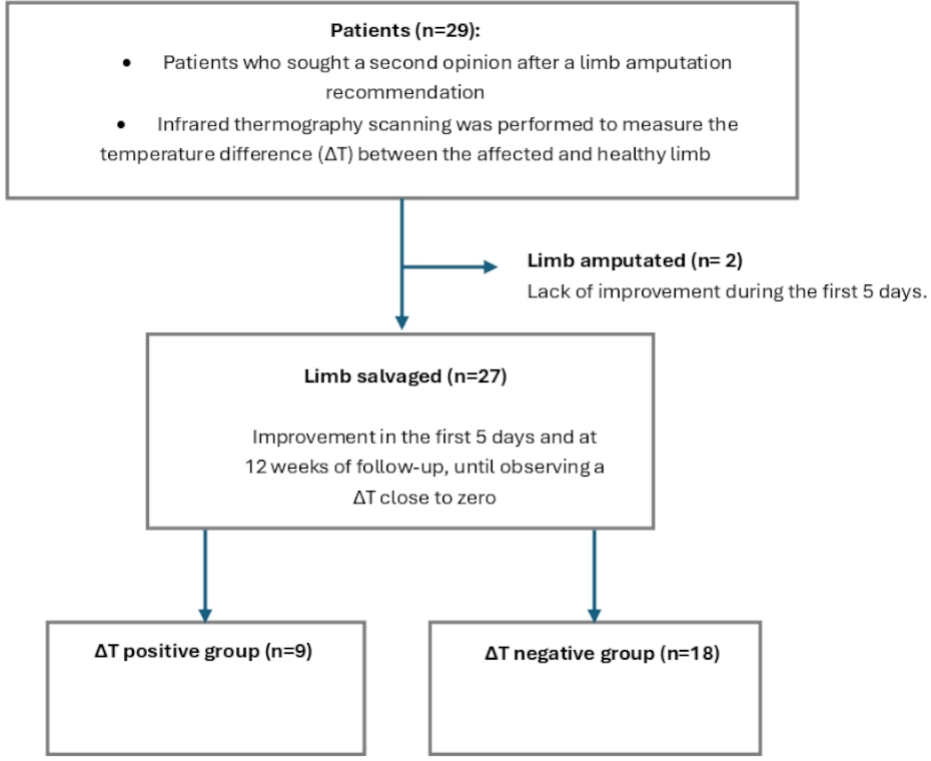
Flowchart of patient inclusion and outcomes in a prospective cohort study of 29 patients at risk of limb amputation evaluated with infrared thermography at Hospital Central “Dr. Ignacio Morones Prieto,” Mexico (Nov 2023-Nov 2024). Among them, 20 patients presented with a negative ΔT, with limb salvage achieved in 18 patients and amputation required in 2 patients.

Over a 12-month period, we included 29 patients with wounds referred for a second opinion regarding limb salvage, with a mean age of 42 (SD 18) years (Figure 1). Seventeen patients were male and 12 were female participants. Twelve patients were evaluated due to trauma, 14 had diabetic foot, 2 had Raynaud disease, and 1 had osteomyelitis. The average wound duration at the time of evaluation was 30 (SD 3) days.

**Figure 2.**
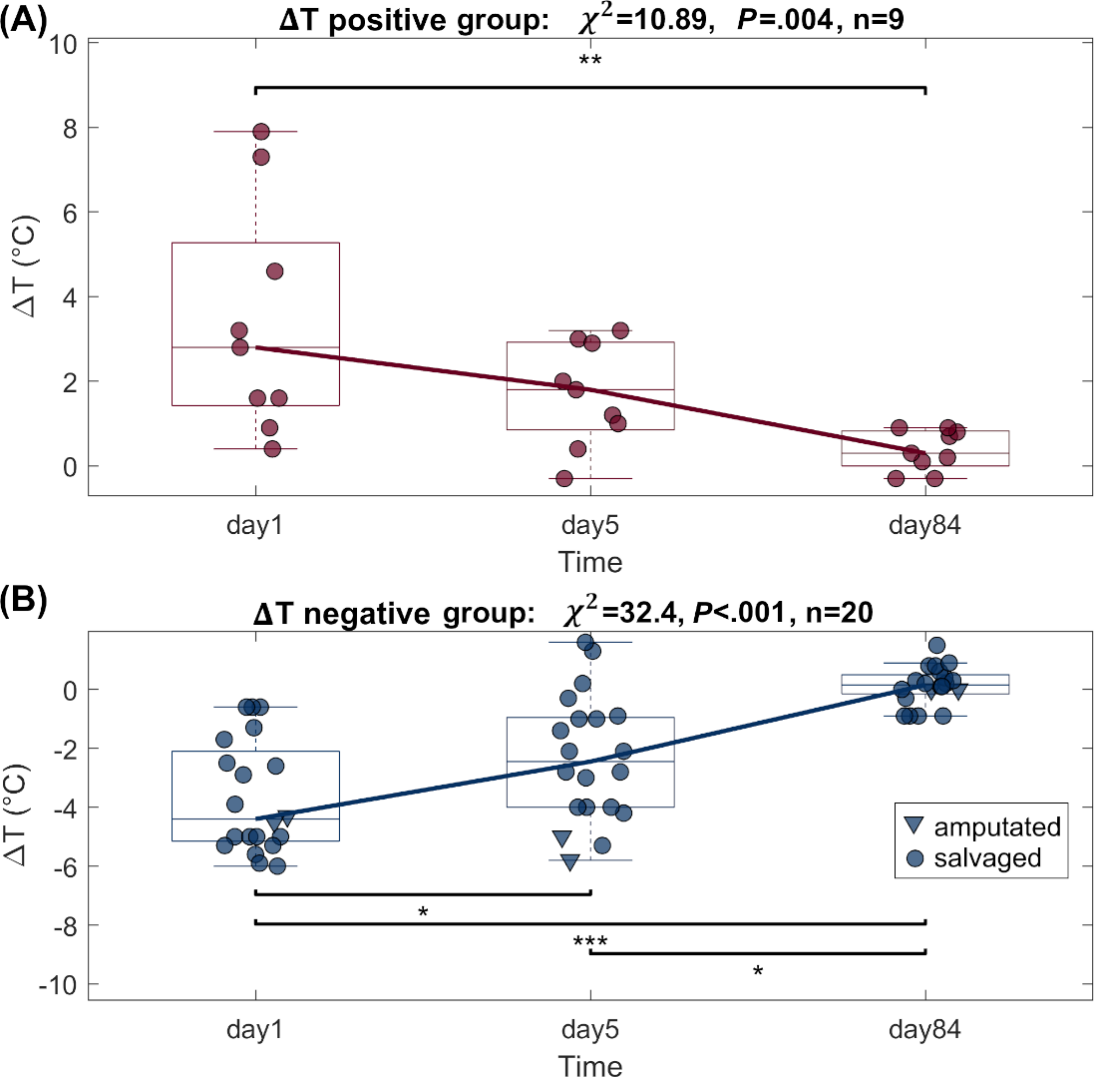
ΔT values of patients at risk of limb amputation, stratified by thermal asymmetry pattern: (A) ΔT-positive group and (B) ΔT-negative group. Each plot shows ΔT values across three time points (Day 1, Day 5, and Day 84). Asterisks indicate levels of statistical significance (*P < .05, **P < .01, ***P < .001). 20 patients presented with a negative ΔT, with limb salvage achieved in 18 cases and amputation required in 2.

Of the 29 patients, 27 limbs were salvaged, and 2 underwent amputation (Table 1). A further subgroup analysis of the salvaged limbs revealed two distinct groups: one with a positive ΔT between the affected limb and healthy limb, and the other with negative ΔT. The Friedman test showed a significant difference across time points for both the positive ΔT group (n=9; *χ*^2^=10.89, *P*=.004) and the negative ΔT group (n=20; *χ*^2^=32.4, *P*<.001) groups. Furthermore, the Mann-Whitney *U* test revealed significant differences between the median ΔT values of both groups on day 1 and day 84 of measurement (*P*<.01). The negative ΔT group presented statistically significant differences between day 1 and day 5, as well as between day 5 and day 84 (Table 2). The temperature difference between the affected limb and the healthy one over time tended toward zero in both groups. (Figure 2).

**Table 1.** Baseline demographic and clinical characteristics of patients at risk of limb amputation, stratified by ΔT^a^ group (positive vs negative).

Variables	Patients (N=27), n (%)	ΔT^a^ positive group (n=9), median (IQR)/n (%)	ΔT negative group (n=18), median (IQR)/n (%)	*P* value^b^
Age (years), n	42.3	42 (18‐67)^c^	42.44 (18-68)^c^	.95^d^
Sex				.45^e^
Male	15 (55.5)	4 (14.8)^f^	11 (40.7)^f^	
Female	12 (44.4)	5 (18.5)^f^	7 (25.9)^f^	
Etiology				.39^g^
Diabetes	12 (44.4)	4 (14.8)^f^	8 (29.6)^f^	
Raynaud-disease	2 (7.4)	0	2 (7.4)^f^	
Osteomyelitis	1 (3.7)	1 (3.7)^f^	0	
Trauma	12 (44.4)	4 (14.8)^f^	8 (29.6)^f^	
Time since onset (days)				.03^e^
≤30	24 (88.8)	25 (21-30)^c^	9 (1-30)^c^	
>30	3 (11.1)	220 (60-320)^c^	–^h^	
Comorbidities	19 (70.3)	2 (7.4)^f^	17 (62.9)^f^	.98^g^
Affected region				.24^g^
Toe	7 (27)	2 (7.4)^f^	5 (18.5)^f^	
Finger	5 (18.5)	1 (3.7)^f^	4 (14.8)^f^	
Foot	9 (33.3)	2 (7.4)^f^	7 (25.9)^f^	
Hand	2 (7.4)	2 (7.4)^f^	0^f^	
Leg	4 (14)	2 (7.4)^f^	2 (7.4)^f^	
WIfI^i^ classification, n (%)				.004^e^
2	1 (3.7)	0^f^	1 (3.7)^f^	
3	7 (25.9)	1 (3.7)^f^	6 (22.2)^f^	
4	4 (14.8)	0^f^	4 (14.8)^f^	

a ΔT: temperature difference.

b *P* values exhibit the comparison analysis between the group of preserved limbs and amputated limbs.

c median (IQR)

d Mann-Whitney *U* Test,

e Fisher Exact Test,

f n (%)

g *χ*2 test.

h Not applicable.

i WIfI: Wound, ischemia, and foot infection.

**Table 2.** ΔT^a^ values in patient ΔT positive and ΔT negative groups.

Time point 1 and Time point 2	Difference in temperature (ΔT), (°C), median (IQR)	*P* value^b^^c^
ΔT positive group		
Day 1 and Day 5	0.78 (0.35 to 1.91)	.30
Day 1 and Day 84	1.56 (0.43 to 2.68)	.003
Day 5 and Day 84	–0.35 (0.78 to 1.91)	.30
ΔT negative group		
Day 1 and Day 5	–1.66 (–0.90 to –0.14)	.01
Day 1 and Day 84	–2.56 (–1.80 to –1.04)	<.001
Day 5 and Day 84	–1.66 (–0.90 to –0.14)	.01

a ΔT: temperature difference.

b *P* values show the temperature differences between the ΔT positive and ΔT negative groups on day 1, day 5, and day 84 of evaluation.

c Post-hoc pairwise test: Wilcoxon-Mann-Whitney test and Bonferroni correction were used for multiple comparisons.

**Figure 3.**
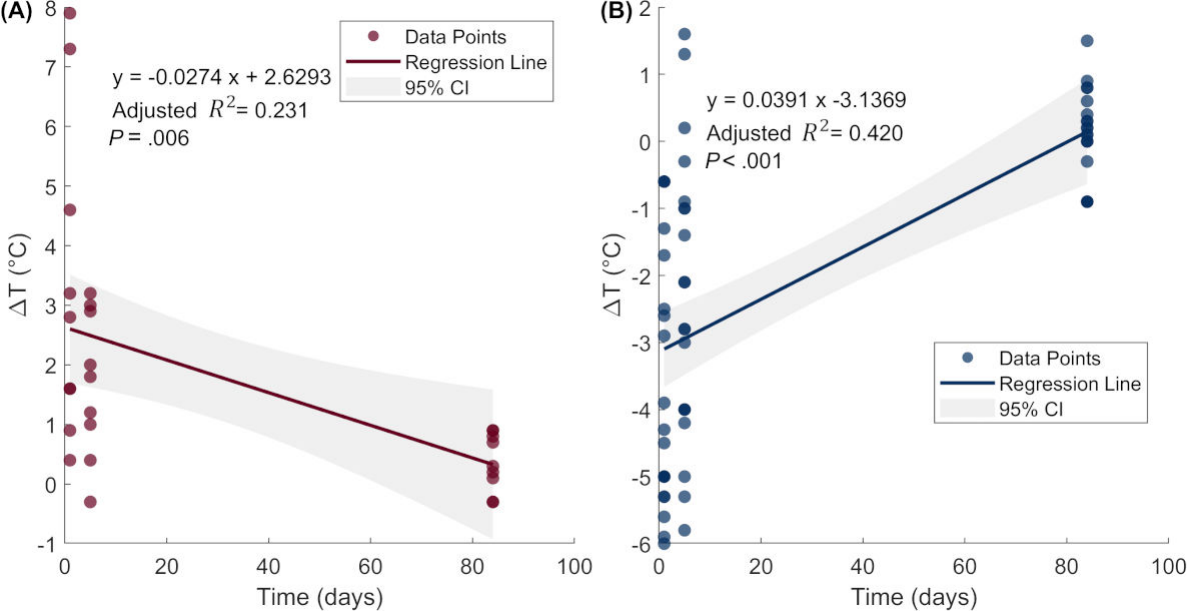
Linear regression of temperature change (ΔT, °C) over time (days) in (A) the ΔT-positive group and (B) the ΔT-negative group. The data points are represented as circles; solid lines indicate the fitted regression lines, and shaded bands represent the 95% CI.

To evaluate the relationship between ΔT and time, linear regression was performed for each group (Figure 3). The regression graph for the positive ΔT group shows a decreasing trend in ΔT values over time, with the following equation:


y=−0.0274x+2.6293

In contrast, the negative ΔT group shows an increasing trend in ΔT values over time:


(y=0.0391x–3.1369)

The two patients who required amputation showed a greater decrease in negative ΔT between days 1 and days 5, in contrast to the other patients in the negative ΔT group, who showed an increased ΔT during the same period.

**Figure 4.**
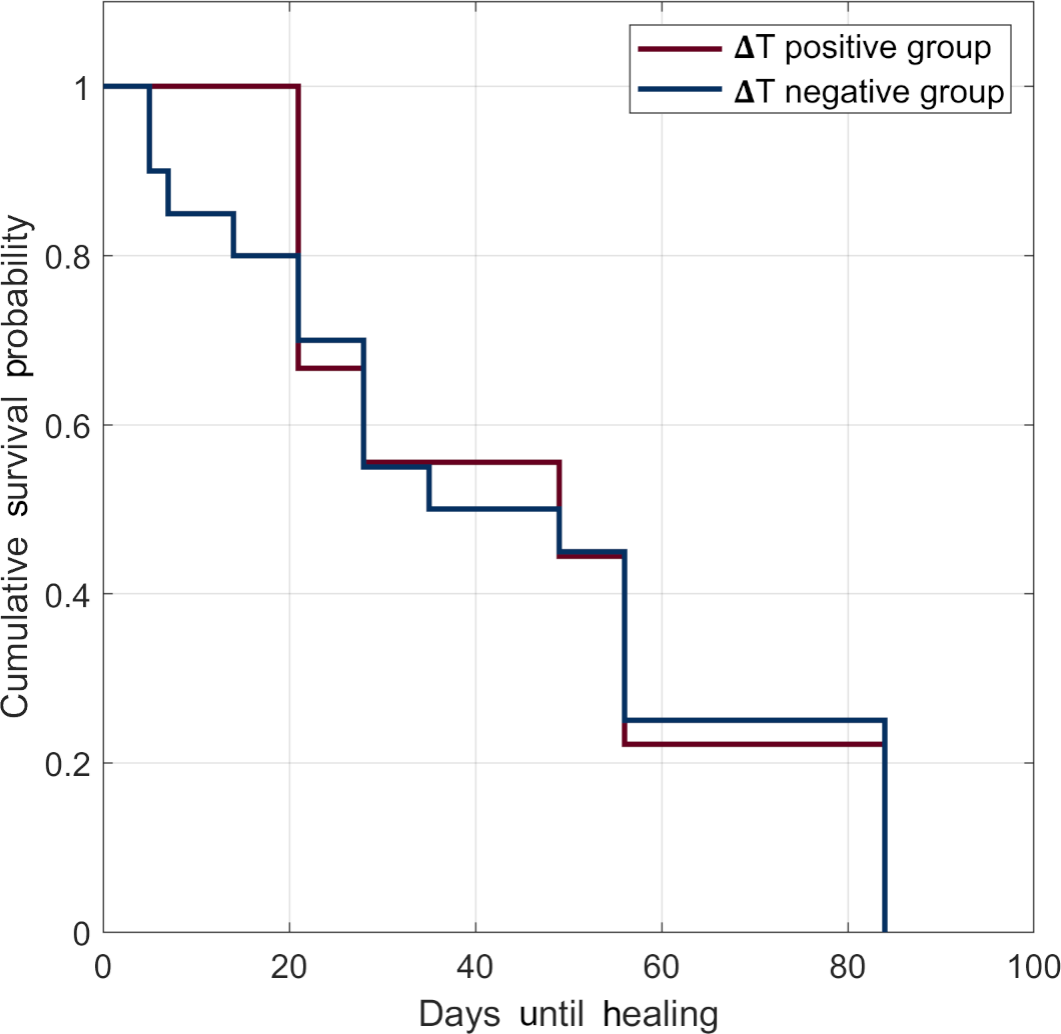
Difference in healing time between ΔT positive and ΔT negative groups using the Kaplan-Meier curve.

Kaplan-Meier analysis using the long-rank test showed no significant difference in survival distributions between the ΔT positive and ΔT negative groups (*P<*.95). Therefore, the survival distributions between both groups were similar (Figure 4).

## Discussion

### Principal Results

**Figure 5.**
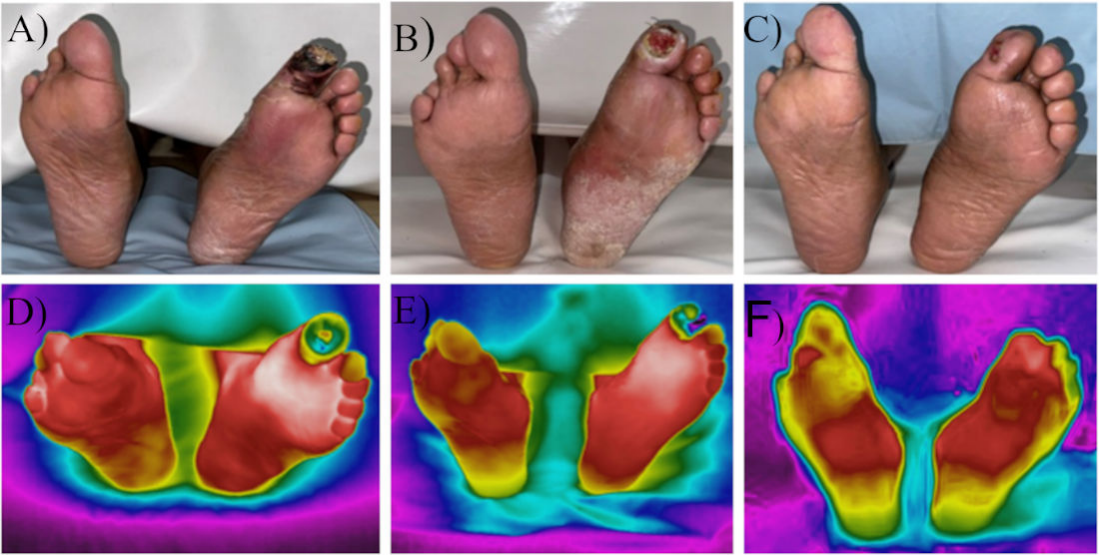
Thermographic assessment of limb salvage. A 67-year-old female patient with a previous history of long-term diabetes and three previous episodes of diabetic foot presented to the Burn Care Unit and Wound Center. (A) She arrived with a 7-day-old wound on the first toe with local necrotic areas. (B) After evaluation, she received treatment with antibiotics, local debridement, and silver wound coverage. (C) Two weeks later the patient was discharged and periodically evaluated until wound closure during follow-up consultations. Areas with the lowest temperatures are displayed in black (D-F).

This study shows the thermographic progression of patients at risk of amputation. It reveals two patterns of temperature, one with an increase and another with decrease in temperature in the regions of interest. The tendency of both groups was toward equalizing the temperature of the healthy limb. The two patients who required amputation showed an initial negative ΔT >5 °C, which worsened by the day 5. Two clinical vignettes, each showing a clinical case from one of the groups, are depicted in Figures5  6.

**Figure 6.**
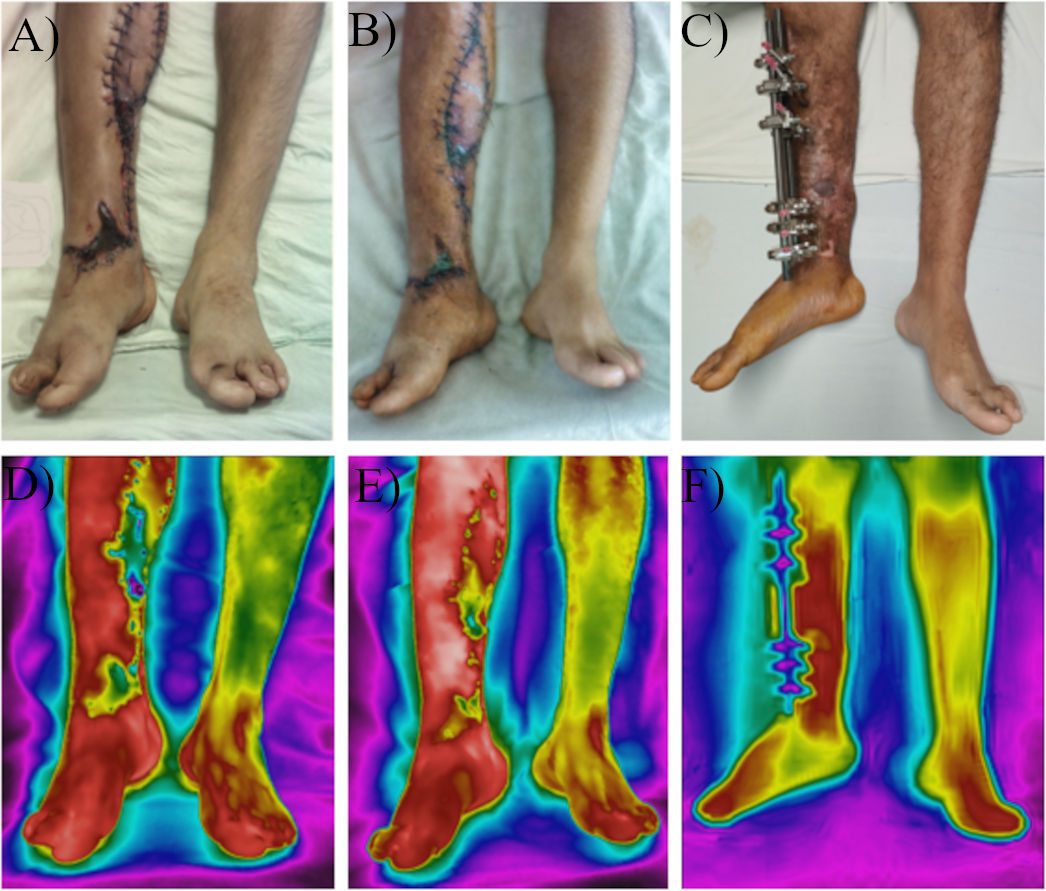
Thermographic assessment of limb salvage. (A) A 31-year-old male patient with a traffic injury on the right lower limb was evaluated after experiencing flap necrosis and wound infection. (B) The patient received treatment with antibiotics, progressive eschar debridement, and silver wound coverage. (C) Two months later, the patient was discharged and periodically evaluated until wound closure during follow-up consultations. Areas with the highest temperatures are displayed in white (D-F).

Previous studies have developed classification systems to support decision-making for amputations, as well as developed methods to stratify the risk of amputations in patients with ulcers. For example, the International Working Group on the Diabetic Foot criteria establishes the risk of ulceration in diabetic patients, while Hüsers et al [6] developed a tool to evaluate amputation risk based on data from the PEDIS (perfusion, extent, depth, infection and sensation) classification for diabetic foot. Prognostic systems including prediction models, offer better assessments than scoring systems by indicating the probability of amputation to physicians. Therefore, adding additional thermographic data to a model could help to improve the actual models. Due to improvements in modern cameras, the production of high-resolution thermal images has allowed the use of thermographic imaging in the detection of diseases related to abnormalities in blood flow such as arterial occlusive disease [1,6].

### Limitations

The sample size and its single-center design are among the main limitations of this work. glycosylated hemoglobin levels, prior interventions, infection severity, and vascular status were not included as variables in the statistical analysis, which may have limited the interpretation of data. The decision to perform an amputation depends on factors beyond temperature measurements. Further studies are required to collect data from a larger cohort of nonamputated patients throughout their clinical evolution, including functional assessment of the affected limb in terms of range of motion, strength, and sensitivity. Additionally, more data is required regarding the observed decrease in temperature values among patients whose limbs were amputated. A longer follow-up study of patients with longer healing times is necessary to determine whether prolonged healing is associated with a reduced life expectancy and poorer prognosis caused by impairment in other organ systems [12,14,15].

### Comparison With Prior Work

Studies on skin microcirculation have established that infrared thermography is equivalent to other methods such as laser Doppler flowmetry, transcutaneous oxygen measurement, and capillaroscopy [4,19,26]. Arteriography is the gold standard method to evaluate blood vessels but is not routinely used due to its invasive nature and the risks associated with radiation and contrast, limiting its application in surgical planning for patients with ischemia [20]. In contrast, the current trend in medicine is to offer personalized and evidence-based care, performed using intravital noninvasive imaging technologies such as thermography. These tools provide valuable data to interdisciplinary teams to enhance the quality of life for patients and providing offloading systems for public health [27]. In future studies, it will be necessary to add more variables to the model such as critical limb ischemia, due to its association with diabetes mellitus and renal insufficiency [28]. Establishing the correlation between temperature, vascular flow, and tissular oxygen impairment requires the incorporation of other techniques such as finger plethysmography, micromyography, noninvasive tissular oxygen measurement, and laser Doppler flowmetry [29]. In the future, thermal data could be used to evaluate the limbs at risk by complementing the existing scores, which is a necessary addition considering that the rates of diabetes mellitus will continue to increase in the future, leading to increased costs and associated complications such as ulcerations, amputations, and deaths; notably 85% of the patients with a lower limb amputation had an ulcer and 47.9% of amputated patients are die within a year [1,30]. Early identification of the need for amputation can reduce these complications, reduce hospital stay and follow-up visits[2]. The global use of infrared thermography as a complementary tool is a feasible approach; it can be implemented not only in cities and large hospitals but also in remote areas and small clinics, as the use of thermal cameras by coupling with smartphones is easy, cheap and requires short-time capacitation sessions for health professionals and students. These results can be interpreted remotely using telemedicine, and can improve the use of resources, referral to a multicenter hospitals, and the medical teams’ response times for limb salvage [23,31].

### Conclusions

Limb salvage was performed using infrared thermography in patients at risk of limb amputation. This method allows comparisons of the thermal difference between the affected and nonaffected contralateral limbs, showing the thermal changes occurring within the treated limb over time. Further studies with more patients should be performed to provide insights into the clinical and progression-related differences between these group of patients.

## Supplementary material

10.2196/69072 Checklist 1 STROBE (Strengthening the Reporting of Observational studies in Epidemiology) checklist

## Abbreviations

MESS: Mangled Extremity Severity Score
WIfI: Wound, ischemia, and foot infection
ΔT: Difference of temperature between two areas of interest
